# A novel gene signature derived from the CXC subfamily of chemokine receptors predicts the prognosis and immune infiltration of patients with lung adenocarcinoma

**DOI:** 10.1097/MD.0000000000030982

**Published:** 2022-10-14

**Authors:** Kun Deng, Shenghua Lin, Zhanyu Xu, Junqi Qin, Liqiang Yuan, Yu Sun, Jiangbo Wei, Tiaozhan Zheng, Zhiwen Zheng, Fanglu Qin, Shikang Li

**Affiliations:** a Department of Thoracic and Cardiovascular Surgery, The First Affiliated Hospital of Guangxi Medical University, Nanning, Guangxi Zhuang Autonomous Region, P. R. China; b Department of Thoracic and Cardiovascular Surgery,The People’s Hospital of Guangxi Zhuang Autonomous Region, Nanning, Guangxi Zhuang Autonomous Region, P. R. China; c Guangxi Medical University, Nanning, Guangxi Zhuang Autonomous Region, P. R. China.

**Keywords:** chemokine receptor CXCR subfamily, gene signature, immune infiltration, lung adenocarcinoma, prognosis

## Abstract

The highly malignant nature of lung adenocarcinoma (LUAD) makes its early diagnosis and prognostic assessment particularly important. However, whether the CXC subfamily of chemokine receptors (CXCR) is involved in the development and prognosis of LUAD remains unclear. Here, differentially expressed genes (DEGs) associated with overall survival (OS) were selected from the cancer genome atlas (TCGA) dataset using univariate Cox analysis and least absolute shrinkage and selection operator (LASSO) regression analysis. Then, a prognostic gene signature was constructed, which was evaluated using Kaplan–Meier curves, receiver operating characteristics curves, nomogram curves, and an external gene expression omnibus (GEO) dataset. Finally, we verified the functions of the genes comprising the signature using the gene expression profiling interactive analysis (GEPIA) and the immune system interaction database (TISIDB) web portals. We constructed a 7-gene signature (*SHC1*, *PRKCD*, *VEGFC*, *RPS6KA1*, *CAT*, *CDC25C*, and *GPI*) that stratified patients into high- and low-risk categories. Notably, the risk score of the signature was a separate and effective predictor for OS (*P* < .001). Patients in the low-risk category had a better prognosis than those in the high-risk category. The receiver operating characteristics and nomogram curves verified the predictive power of the signature. Moreover, in both categories, biological processes and pathways associated with cell migration were enriched. Immune infiltration statuses differed between the 2 risk categories. Critically, the results from the GEPIA and TISIDB web portals indicated that the expression of the 7-gene signature was associated with survival, clinical stage, and immune subtypes of LUAD patients. We identified a CXCR-related gene signature that could assess prognosis and provide a reference for the diagnosis and treatment of LUAD.

## 1. Introduction

Lung cancer is a great threat to the health and lives of the population because of its high malignancy, fastest rising incidence and mortality rates.^[1,2]^ Of all histological subtypes, lung adenocarcinoma (LUAD) has the highest incidence.^[3]^ Thus far, advancements in understanding the potential mechanisms associated with LUAD have led to the development of multiple targeted drugs, which have been greatly beneficial in improving the prognosis of patients with LUAD.^[4]^ However, patients inevitably develop adverse reactions, drug resistance, and other complications during drug treatment programs such as gefitinib, erlotinib, and bevacizumab.^[4,5]^ Consequently, the focus has been on improving the prognosis of LUAD patients and developing new target drugs. The establishment of a prognosis-associated gene signature is urgent in the search for tumor-related biomarkers.

Chemokines and their receptors constitute of a large category of small-secreted proteins that are necessary during the execution of immune system function.^[6–8]^ They are also key mediators of cancer-associated inflammation, as they are present at the tumor site and can therefore directly influence the proliferation, infiltration, and metastasis of cancer cells.^[9,10]^ To date, over 50 human-related chemokines have been identified, and could be divided into 4 subfamilies on the basis of relative locations of their cysteine residues: C, CC, CXC, and CX3C.^[11,12]^ In most cases, chemokine-mediated signaling pathways are only activated when chemokines selectively bind to receptors expressed on the target cells’ surfaces.^[11,13,14]^ At present, many chemokines and their receptor antagonists have been approved.^[11,15]^ For example, plerixafor, a small molecule CXCR4 antagonist, can increase the ratio of stem/progenitor cells in peripheral blood. Maraviroc, a CCR5 antagonist, is used in anti-HIV therapy. Additional drug candidates, which include CCR5, CXCR4, and CCR2/CCR5 dual antagonists such as leronlimab, motixafortide, and cenicriviroc, respectively, are undergoing phase 3 clinical experiments.^[11]^ As the largest class in the chemokine receptor family,^[16]^ the CXC subfamily is the most promising. Many chemokine-related genes have potential in the development of more targeted drugs that can ameliorate the prognosis of LUAD patients. However, whether CXC receptors (CXCRs) are related to the development and prognosis of LUAD and whether it can be used as a therapeutic target remains unclear.

Here, we used 2 common public databases (the cancer genome atlas [TCGA] and gene expression omnibus [GEO]) to obtain mRNA expression and relevant clinical data of LUAD patients. We then applied univariate analysis and east absolute shrinkage and selection operator (LASSO) Cox regression analysis to data from the TCGA dataset to identify a prognostic gene signature comprising of CXCR-related differentially expressed genes (DEGs) and verified through a GEO dataset. Afterwards, we applied Gene ontology (GO) and Kyoto Encyclopedia of Genes and Genomes (KEGG) enrichment analyses to search for potential mechanisms. Finally, we verified the nomogram’s prognostic potential using the gene expression profiling interactive analysis (GEPIA) web-based tool and explored the correlation between its signature genes with immune subtypes using the immune system interaction database (TISIDB) web portal. It is worth mentioning that the TISIDB platform was developed to promote comprehensive research on tumor-immune interactions.^[17]^

## 2. Materials and methods

### 2.1. Data preparation and pre-processing

CXCR-related genes (n = 927) were accessed from the GeneCards website (http://www.genecards.org/).^[18]^ The transcriptome information and relevant clinical data for 551 LUAD patients, which were used to identify the prognostic gene signature, were accessed from the TCGA website (https://portal.gdc.cancer.gov/repository/). For verification, the mRNA expression data profiling by array and clinical data of 163 samples (GPL7015, GSE11969) were obtained from the GEO database (https://www.ncbi.nlm.nih.gov/geo/). We discarded samples with unknown clinical characteristics or that had survival times under 30 days. We also took the log2 logarithm for the TCGA dataset and used the “sva”^[19]^ R package to identify the intersecting genes and to normalize mRNA expression profiles of the 2 datasets from the TCGA and GEO databases. Both TCGA and GEO’s data resources are open to the public. Furthermore, our research adheres to the TCGA and GEO data access and publication requirements. The data used in this study were obtained from public databases such as TCGA and GEO, and no human or animal experiments were involved. Therefore, ethical approval from the Ethics Committee of Guangxi Medical University is not required.

### 2.2. Construction and verification of a prognostic CXCR-related gene signature

Perl software was used to merge transcriptome and clinical data. We used the “limma”^[20]^ R package to distinguish DEGs between tumor specimens and neighboring normal specimens (false discovery rate < 0.001) in the TCGA dataset. Univariate Cox analysis was applied to screen prognostic CXCR-related DEGs (*P* < .001). Then, we carried out LASSO Cox regression^[21]^ to build a prognostic gene signature. The formula below was used to determine the risk score:


$$\begin{array}{l} riskscore=\underset{i=1}{\overset{n}{\sum }}\;Expi\times Coefi \end{array}$$

where *n* means gene numbers in the signature, *Expi* represents the expression level of each signature gene, and *Coefi* represents the LASSO regression coefficient. Considering that the risk score of each patient was not normally distributed, we chose to divide the patients into 2 risk (high-risk and low-risk) categories using the median risk value. This way we obtained as many patients in both risk categories and could further compare their overall survival (OS) to verify whether there was a difference in prognosis between the 2 risk categories.

In addition, we used the STRING database (https://string-db.org/cgi/input.pl) to create an interaction network with the intersecting prognostic DEGs according to the expression of signature genes. We also draw risk heat maps to represent the association of signature genes with risk categories. We defined the range of expression levels of these genes as 0 to 2.5, with red representing high expression and green representing low expression, and the color change from left to right represents the change in expression levels of genes in-high and low-risk categories. To evaluate the distribution statuses between the high- and low-risk patient categories, principal component analysis (PCA) and t-distributed stochastic neighbor embedding (t-SNE) were performed using the “ggplot2”^[22]^ and “Rtsne” R packages.^[23]^ We used the “survival” and “survminer”^[24]^ R packages to compare the difference in OS between the 2 risk categories and to plot survival curves. Moreover, we used the “survivalROC”^[25]^ R package to perform receiver operating characteristics curve analyses to assess the gene signature’s predictive performance. The “rms” R package was used to set up a nomogram that best predicted the prognosis of LUAD patients.^[26]^

### 2.3. GO enrichment, KEGG enrichment, and immune infiltration analysis

**Figure 1.**
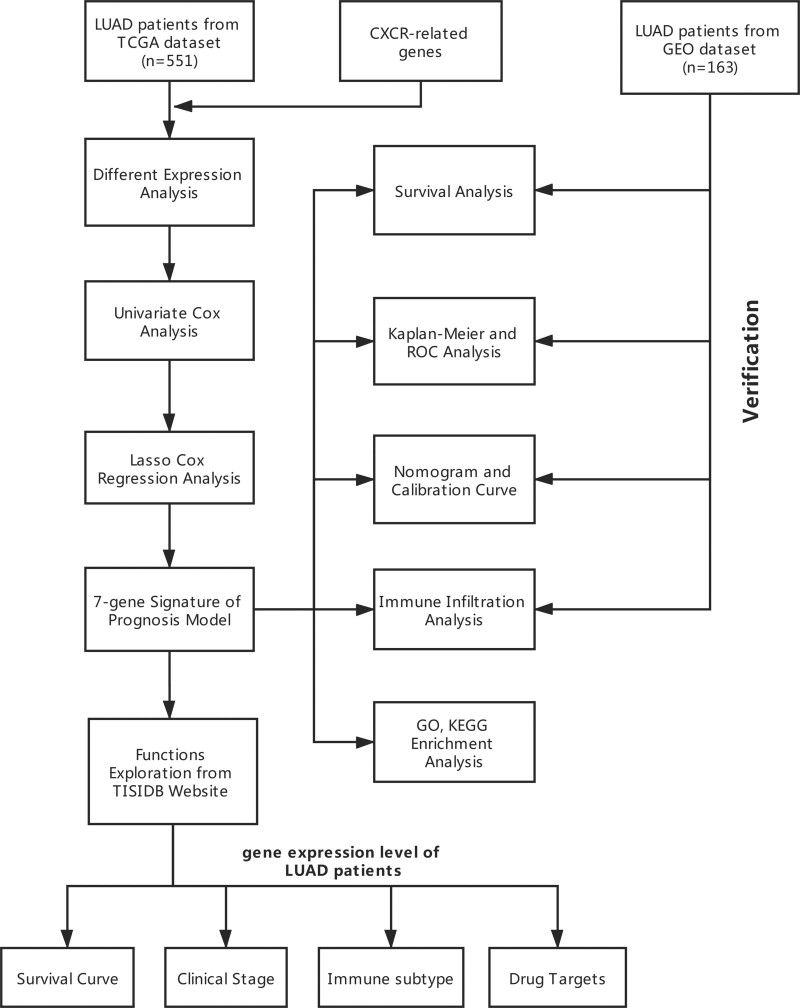
The research roadmap of the present study.

GO (*P* < .05, *q* < 0.05) and KEGG (*P* < .05) enrichment analyses based on the DEGs were conducted between the 2 risk categories with the “clusterProfiler”^[27]^ R package. The single-sample gene set enrichment analysis (ssGSEA) and “gsva”^[28]^ R package were used to measure the infiltration scores of 16 immune cells and 13 immune function pathways (As shown in Fig. 1C-F).^[29]^

### 2.4. Function exploration in the GEPIA and TISIDB web portals

The GEPIA website (http://gepia.cancer-pku.cn/) contains an immense amount of RNA sequencing data from the TCGA and other databases.^[30]^ To verify the prognostic potential of our signature genes in LUAD patients, we performed survival analysis and clinical staging according to the expression of each gene. Taking advantage of the powerful features of the TISIDB platform (http://cis.hku.hk/TISIDB/), we explored the correlation among the expression levels of the signature genes in LUAD patients with immune subtypes and drug targets.

### 2.5. Statistical analysis

Student *t* test was applied to distinguish DEGs between tumor specimens and neighboring normal specimens. The Chi-squared test was applied to compare relative differences. The ssGSEA scores of immune cells or functional pathways were compared between the high- and low-risk patients using the Mann-Whitney test with the *P* values adjusted using the Benjamini-Hochberg procedure. The log-rank test was applied to compare the OS of the high- and low-risk patients as derived from the Kaplan–Meier analyses. All statistical analyses were conducted using R (version 4.0.2) or SPSS (version 26.0) software. If not specifically mentioned, statistical significance was defined as *P* < .05.

## 3. Results

**Figure 2.**
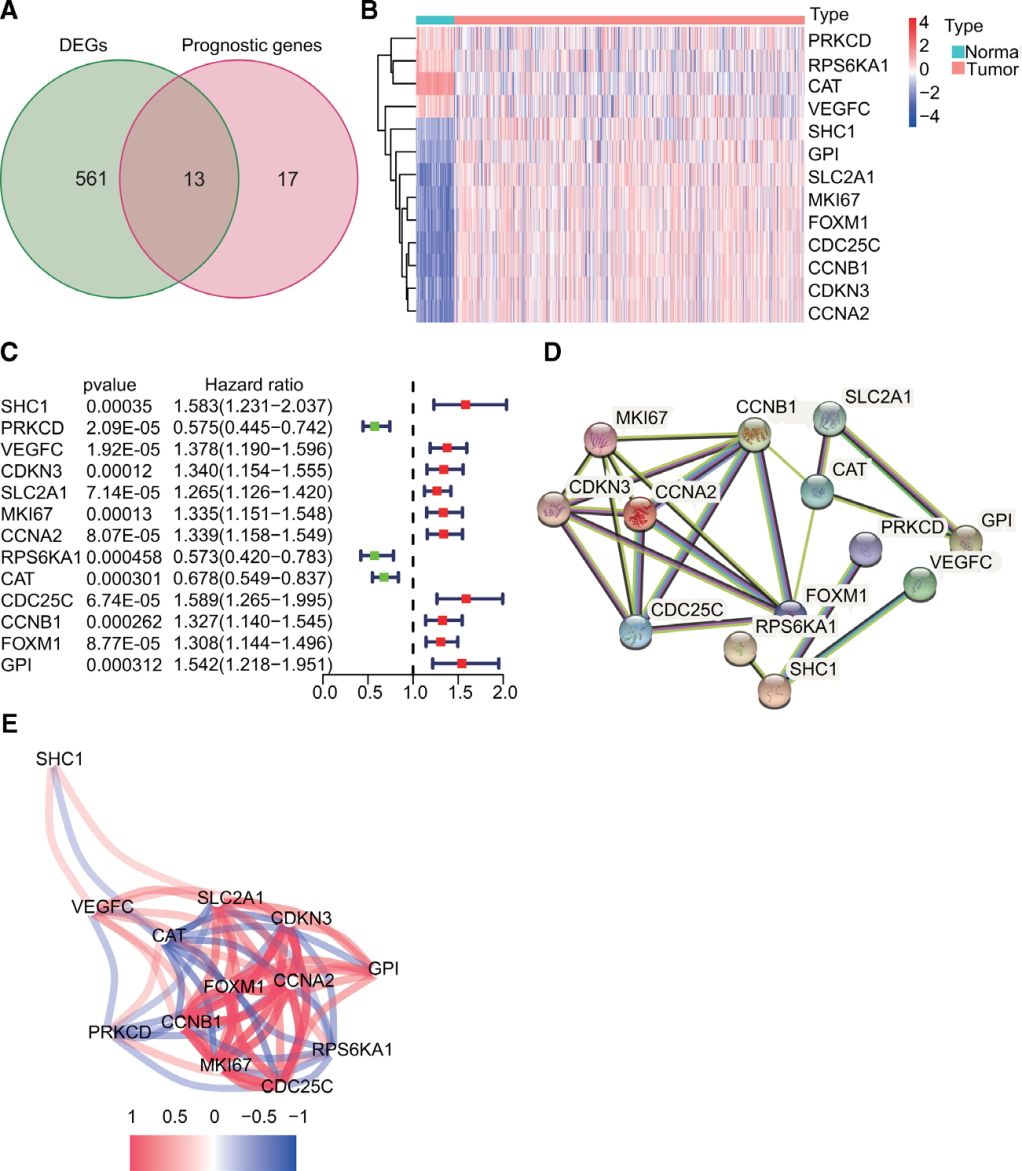
Identification of prognostic CXCR-associated differentially expressed genes (PDEGs) in The Cancer Genome Atlas (TCGA) dataset. (A) Venn diagram for identifying the 13 PDEGs. (B) Heat map showing high (red) and low (blue) levels of expression of the PDEGs. Nine genes were upregulated in tumor tissues, while the *PRKCD*, *RPS6KA1*, *CAT*, and *VEGFC* genes were downregulated. (C) Forest map showing high (red) and low (green) expression of the PDEGs. *PRKCD*, *RPS6KA1*, and *CAT* were protective genes, while the others were risk genes. (D) Protein–protein interaction network of the PDEGs downloaded from the STRING website. *RPS6KA1*, *CDC25C*, *CCNA2*, *CAT*, and *SHC1* were central genes. (E) The correlation network of PDEGs.

The research roadmap of our study is presented in Figure 2. In total, data from 466 and 90 LUAD patients from the TCGA (n = 551) and GEO datasets (n = 163) was included.

### 3.1. Recognization of prognostic CXCR-associated DEGs in the TCGA dataset

**Figure 3.**
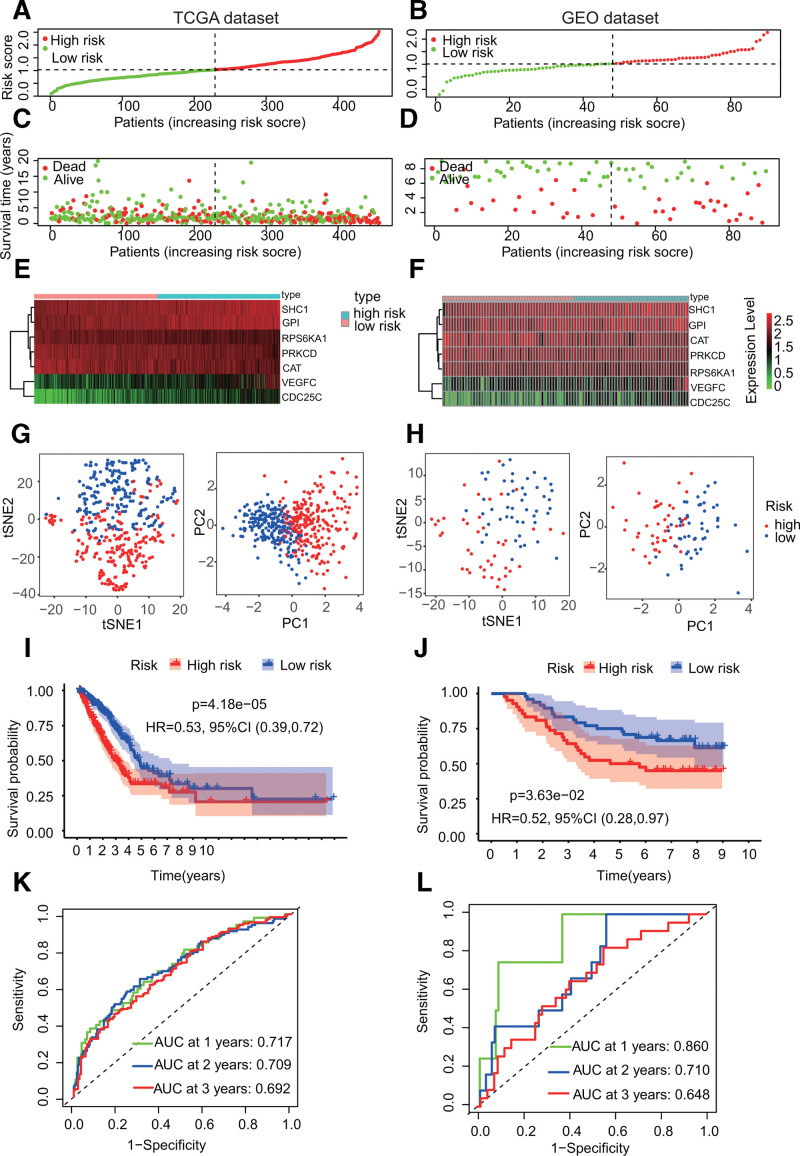
Establishment and performance validation of a prognostic gene signature. (A) The risk curve demonstrated that patients can be divided into high-risk or low-risk categories according to the median value of the risk score in the TCGA dataset. (B) The risk curve from the GEO dataset. (C) The survival status chart from the TCGA dataset demonstrated that patients in the high-risk category had higher mortality rates, while those in the low-risk category were the opposite. (D) The survival status chart from the GEO dataset. (E) The risk heatmap indicates *SHC1*, *GPI*, *VEGFC*, and *CDC25C* are high-risk genes, while *RPS6KA1, PRKCD*, and *CAT* were low-risk genes in the TCGA dataset. (F) The risk heatmap from the GEO dataset. (G) PCA and t-SNE analysis in the TCGA dataset showed that the patients in the 2 risk categories were classified as 2 distribution statuses. (H) PCA and t-SNE analysis in the GEO dataset. I. Kaplan–Meier curves for the OS of LUAD patients in the high- and low-risk categories in the TCGA dataset, *P* < .001. (J) Kaplan–Meier curves in the GEO dataset. (K) Receiver operating characteristic (ROC) curves demonstrated the prognostic value of the risk score in the TCGA dataset. (L) ROC curves in the GEO dataset. TCGA = The Cancer Genome Atlas.

Following differential expression analysis, we found that over half of the CXCR-related genes (574/927, 61.92%) were differentially expressed between tumor and paracancerous tissues (false discovery rate < 0.001). Thirteen of these DEGs were associated with OS as detected by univariate Cox regression analysis (*P* < .001, Fig. 3A). Nine genes were upregulated in tumor tissues, while the *PRKCD*, *RPS6KA1*, *CAT*, and *VEGFC* genes were downregulated (Fig. 3B). Forest plots indicated that *PRKCD*, *RPS6KA1*, and *CAT* were protective genes, while the others were risk genes (Fig. 3C). The interaction network of the 13 DEGs demonstrated that *RPS6KA1*, *CDC25C*, *CCNA2*, *CAT*, and *SHC1* were central genes (Fig. 3D–E).

### 3.2. Establishment of a prognostic gene signature using the TCGA dataset followed by performance verification in the GEO dataset

**Figure 4.**
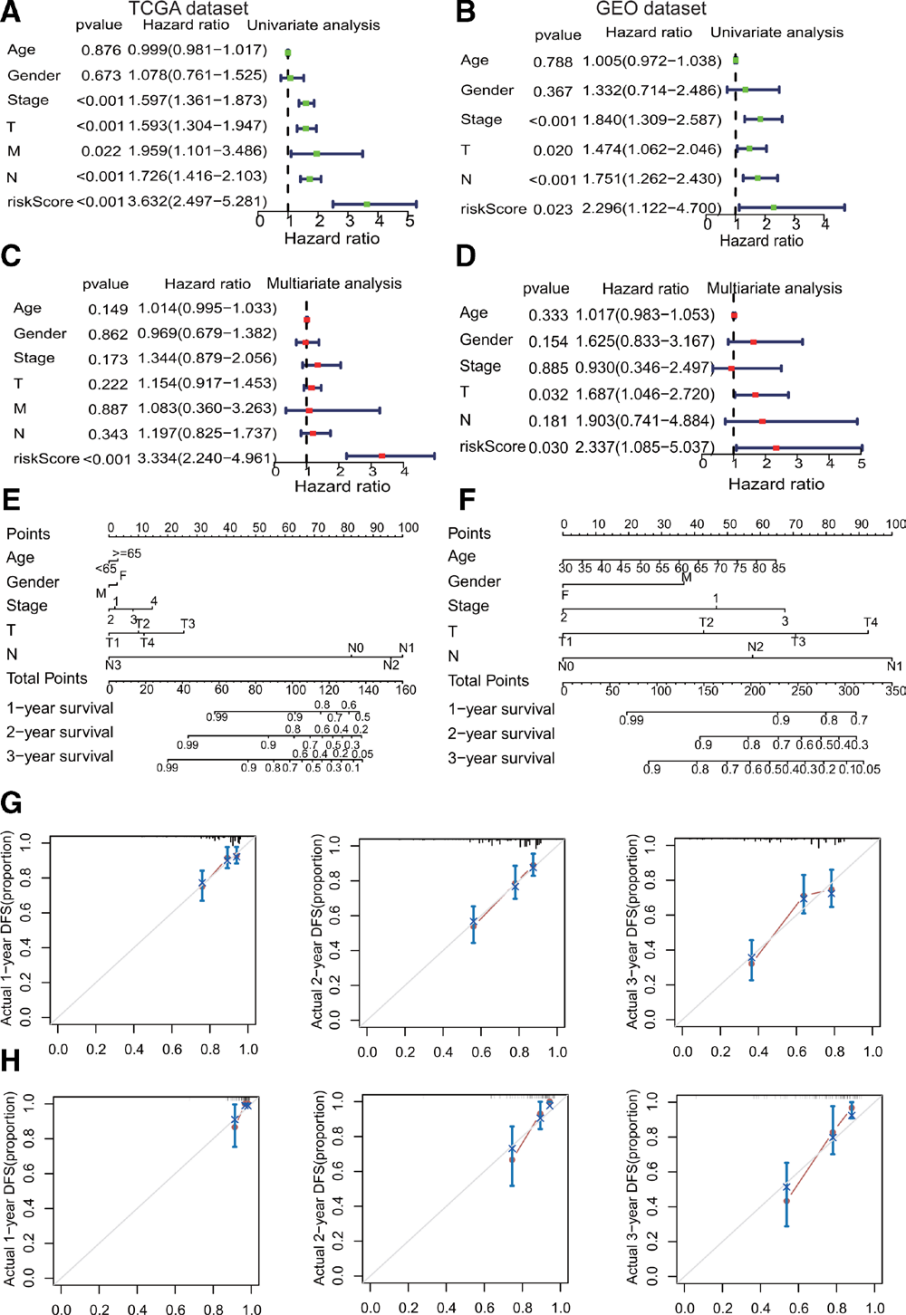
Independent prognostic analysis of the 7-gene signature and construction of the nomogram. (A and B) The results of the univariate Cox regression analyses in the TCGA and GEO datasets. (C and D). The results of the multivariate Cox regression analyses in the TCGA and GEO datasets. (E) Prognostic nomograms to predict the 1, 2, and 3-year disease-free survival (DFS) of LUAD patients in the TCGA dataset. (F) Prognostic nomograms to predict the 1, 2, and 3-year DFS of LUAD patients in the GEO dataset. (G) Validation of calibration curves for predicting DFS values from the nomogram in the TCGA dataset. (H) Validation of calibration curves for predicting DFS values from the nomogram in the GEO dataset. TCGA = The Cancer Genome Atlas.

A prognostic signature was established via LASSO Cox regression using the expression data of the 13 prognostic DEGs. Then, a 7-gene signature was identified using optimal λ values. LASSO regression coefficients were shown in Table 1. Based on the median critical value, patients in the TCGA dataset were classified as high- and low-risk categories (Fig. 4A). The survival analyses revealed that patients belonging to high-risk category had slightly worse OS than those in the low-risk category (Fig. 4C). The risk heatmap indicated that from left to right (that is, from the low-risk category to the high-risk category), the expression levels of *SHC1*, *GPI*, *VEGFC*, and *CDC25C* were increased, so they were all high-risk genes, while the expression levels of *RPS6KA1, PRKCD*, and *CAT* were reduced, so they were low-risk genes (Fig. 4E). The PCA and t-SNE analyses demonstrated that the patients in 2 risk categories were divided into 2 distribution statuses (Fig. 4G). Moreover, the Kaplan–Meier curves demonstrated that patients in the low-risk category had slightly higher OS than those in the high-risk category (Fig. 4I, *P* = 4.18e − 05). The predictive accuracy of risk score for OS was assessed by receiver operating characteristics curves, with the area under the curve reaching 0.717, 0.709, and 0.692 at 1, 2, and 3 years, respectively (Fig. 4K).

**Table 1:** Coefficient of 7-gene signature.

Gene	Coefficient
SHC1	0.285078
PRKCD	-0.26271
VEGFC	0.182141
RPS6KA1	-0.12576
CAT	-0.01815
CDC25C	0.235979
GPI	0.174824

To verify the accuracy of this gene signature established from the TCGA dataset, patients in the GEO dataset were divided into 2 different risk categories according to the median values calculated by the same formula as the TCGA dataset (Fig. 4B). Similarly, survival analysis for the GEO dataset demonstrated that patients in the low-risk category had a slightly higher OS than those in the high-risk category (Fig. 4D). The risk heatmap in the GEO dataset was consistent with that from the TCGA dataset (Fig. 4F). Additionally, the PCA and t-SNE analyses showed that patients in the 2 risk categories were divided into different distribution statuses (Fig. 4H). Similarly, the Kaplan–Meier curves confirmed the prognostic signature’s ability in predicting survival (Fig. 4J, *P* = .0363). Finally, the area under the curve of the 7-gene signature reached 0.860, 0.710, and 0.648 at 1, 2, and 3 years, respectively (Fig. 4L). These results demonstrated the power of our gene signature in predicting prognostic survival of LUAD patients.

### 3.3. Independent prognostic analysis of the 7-gene signature and construction of the nomogram using the TCGA and GEO datasets

**Figure 5.**
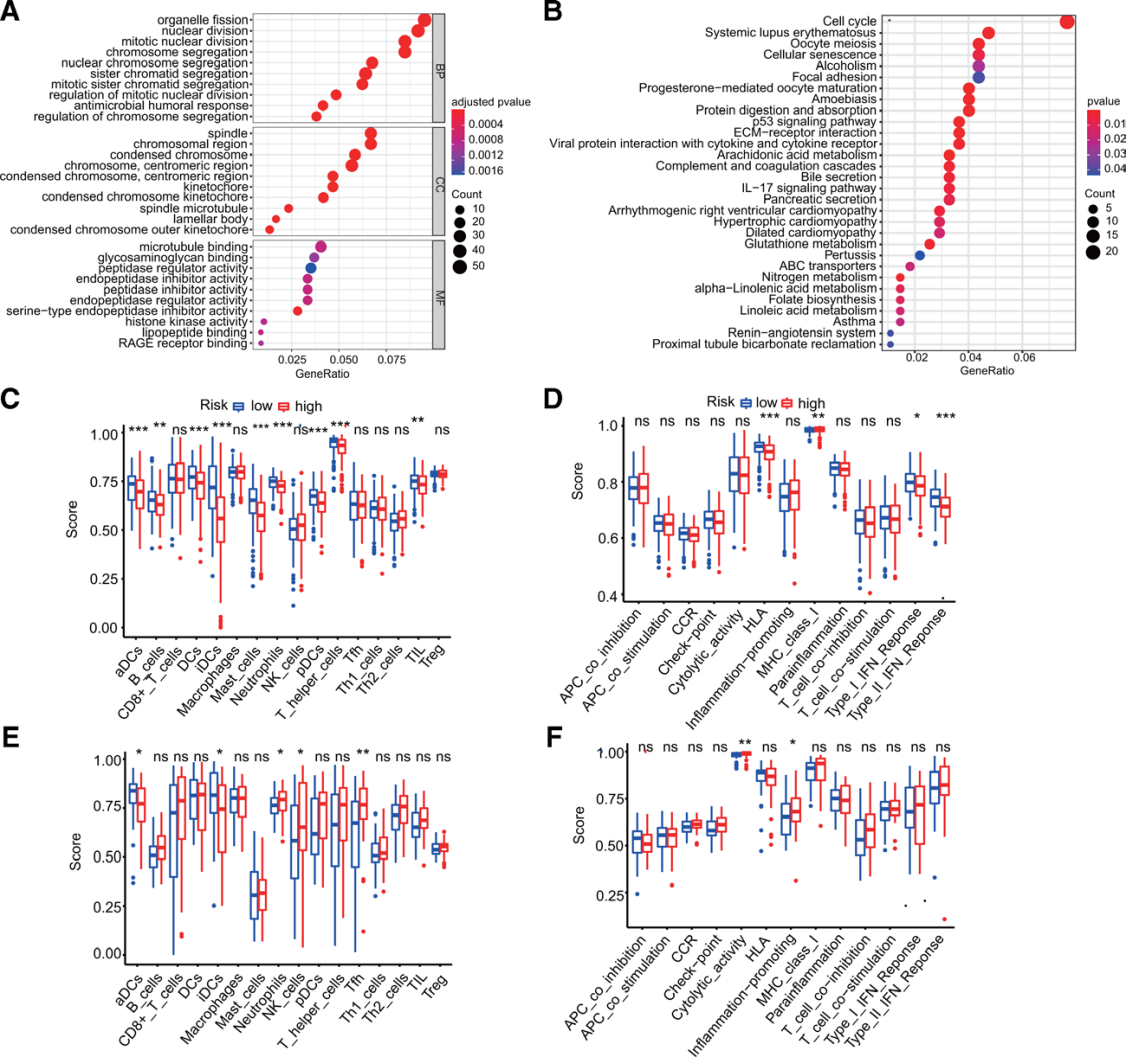
GO, KEGG enrichment, and immune infiltration analysis. (A) Bubble plots from the GO enrichment analysis indicating the most significant pathways in the TCGA dataset (adjusted *P* value < .05). (B) Bubble plots from the KEGG enrichment analysis indicating the most significant pathways in the TCGA dataset (*P* value < .05). (C–F) Box plots related to immune cells (C, E) and immune functions (D, F) in the TCGA (C, D) and GEO (E, F) datasets were obtained by comparing ssGSEA scores between the different risk categories (16 types of immune cells include: aDCs, B_cells, CD8+_T_cells, DCs, iDCs, Macrophages, Mast cells, Neutrophils, NK cells, pDCs, T helper cells, Tfh, Th1cells, Th2 cells, TIL, and Treg. And 13 immune function pathways include: APC co inhibition, APC co stimulation, CCR, Check-point, Cytolytic activity, HLA, Inflammation-promoting, MHC class I, Parainflammation, T cell co-inhibition, T cell co-stimulation, Type I IFN Response, and Type II IFN Response. Statistical significance: ns = not significant; *P < .05; **P < .01; ***p < .001).

Univariate and multivariate Cox regression analyses were applied to examine if risk score can predict OS. Unsurprisingly, the univariate Cox regression analysis demonstrated that risk scores were closely connected with OS in both the TCGA and GEO datasets (TCGA dataset: hazard ratio [HR] = 3.632 [2.497–5.281], *P* < .001, Fig. 5A; GEO dataset: HR = 2.296 [1.122–4.700], *P* = .023, Fig. 5B). The risk score remained an independent predictor of OS in the multivariate Cox regression after other confounding variables were removed (TCGA dataset: HR = 3.334 [2.240 − 4.961], *P* < .001, Fig. 5C; GEO dataset: HR = 2.337 [1.085 − 5.037], *P* = .030; Fig. 5D). The prognostic nomogram predicting disease free survival at 1, 2, and 3 years was created using stepwise Cox regression models derived from patients with complete clinical data from the TCGA (Fig. 5E) and GEO datasets (Fig. 5F). The parameters listed in the nomogram included: age, gender, stage, T-stage, and N-stage. The calibration curve indicated excellent performance of the nomogram in predicting the disease-free survival of LUAD patients in the TCGA (Fig. 5G) and GEO datasets (Fig. 5H).

### 3.4. GO enrichment, KEGG enrichment, and immune infiltration analysis in the TCGA and GEO datasets

To explore the biological functions and pathways relevant to risk scores, GO and KEGG enrichment were performed on the DEGs from the TCGA dataset in the high- and low-risk patients. Unsurprisingly, DEGs were enriched for several molecular functions associated to cell migration, such as microtubule binding pathways and peptidase regulator activity (Fig. 1A). In addition, DEGs were remarkably enriched in processes of nuclear division (Fig. 1A), including nuclear division pathways, regulation of chromosome segregation, condensed chromosomal mitochondria, and external mitochondria (Fig. 1A). The KEGG enrichment analysis indicated that cell cycle and migration pathways were enriched (Fig. 1B), especially extracellular matrix-receptor interaction and the p53 signaling pathway. Furthermore, the DEGs were enriched for systemic lupus erythematosus, dilated cardiomyopathy, amebiasis, and many other diseases (Fig. 1B). Most notably, we also found that CXCR-related DEGs are involved in the IL-17 signaling pathway and the P53 signaling pathway (Fig. 1B), which in turn affect the progression of LUAD.

To further investigate the relationship between risk score and immune status, ssGSEA was applied to measure the enrichment scores of various immune cell subpopulations and their related functions and pathways. The low- and high-risk patients in the TCGA dataset (all adjusted *p*’s < 0.05; Fig. 1C) significantly differed in the content of the antigen presentation process, including scores for dendritic cells (DC), activated DCs, B cells, mast cells, neutrophils, immature DCs, plasmacytoid DCs, T helper cells, tumor-infiltrating lymphocytes, human leukocyte antigen, major histocompatibility complex class I molecules, type II interferon (IFN) responses, and type I IFN responses. More specifically, the high-risk category had lower scores for type II IFN responses, type I IFN responses, and human leukocyte antigen, while major histocompatibility complex class I had the opposite effect (adjusted *P* < .05, Fig. 1D). The differences in cytolytic activity and pro-inflammatory effects between the 2 risk categories were verified in the GEO cohort (adjusted *p*’s < 0.05, Fig. 1F). In both the TCGA and GEO cohorts, the activated DC, immature DC, and neutrophil ratings statistically differed the most between the 2 risk categories (Fig. 1C and E). These results suggested that the immune infiltration status also differed between high and low risk categories according to our signature risk score, which could inform the subsequent treatment of LUAD patients.

### 3.5. Validation of the 7-gene signature using the GEPIA and TISIDB web tools

**Figure 6.**
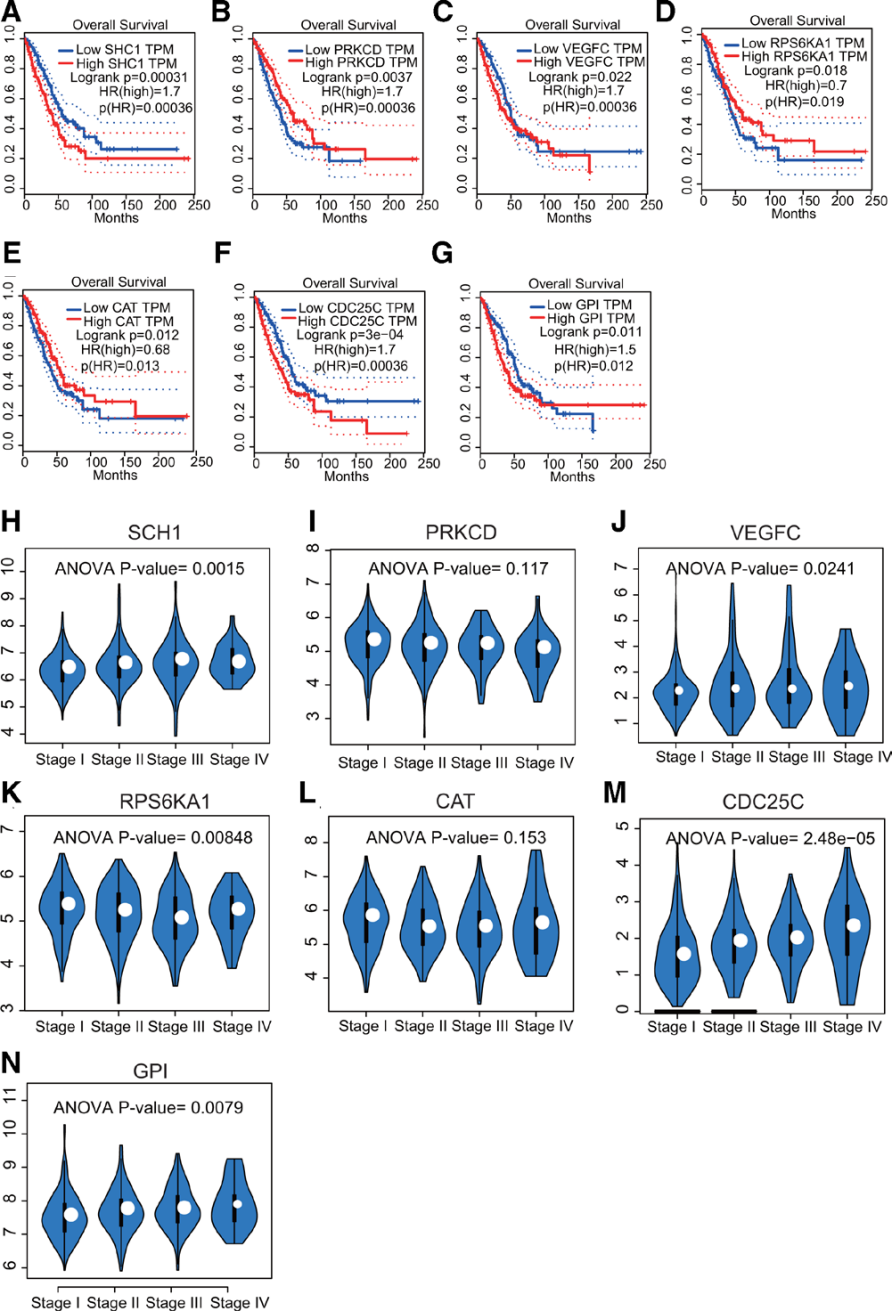
Validation of the prognostic potential of the 7-gene signature using the GEPIA website. (A–G) The Kaplan–Meier curves predicted the OS of LUAD patients according to the transcripts per million (TPM) of the signature genes. The expression levels of all 7 genes in the signature were associated with OS in LUAD patients (*P* < .05). (H–N) The relationship between the expression levels of these genes and clinical stages in LUAD patients. Except for PRKCD (*P* = .117) and CAT (*P* = .153), the expression levels of the remaining 5 genes were significantly correlated with clinical stage of LUAD patients.

The results from the GEPIA web tool indicated that the expression of all these 7 genes in the signature were closely related to the OS (Fig. 6A–G, all *p*’s < 0.05) and clinical stages (Fig. 6H–N, except *PRKCD*, *P* = .117 and *CAT*, *P* = .153) of the LUAD patients.

**Figure 7.**
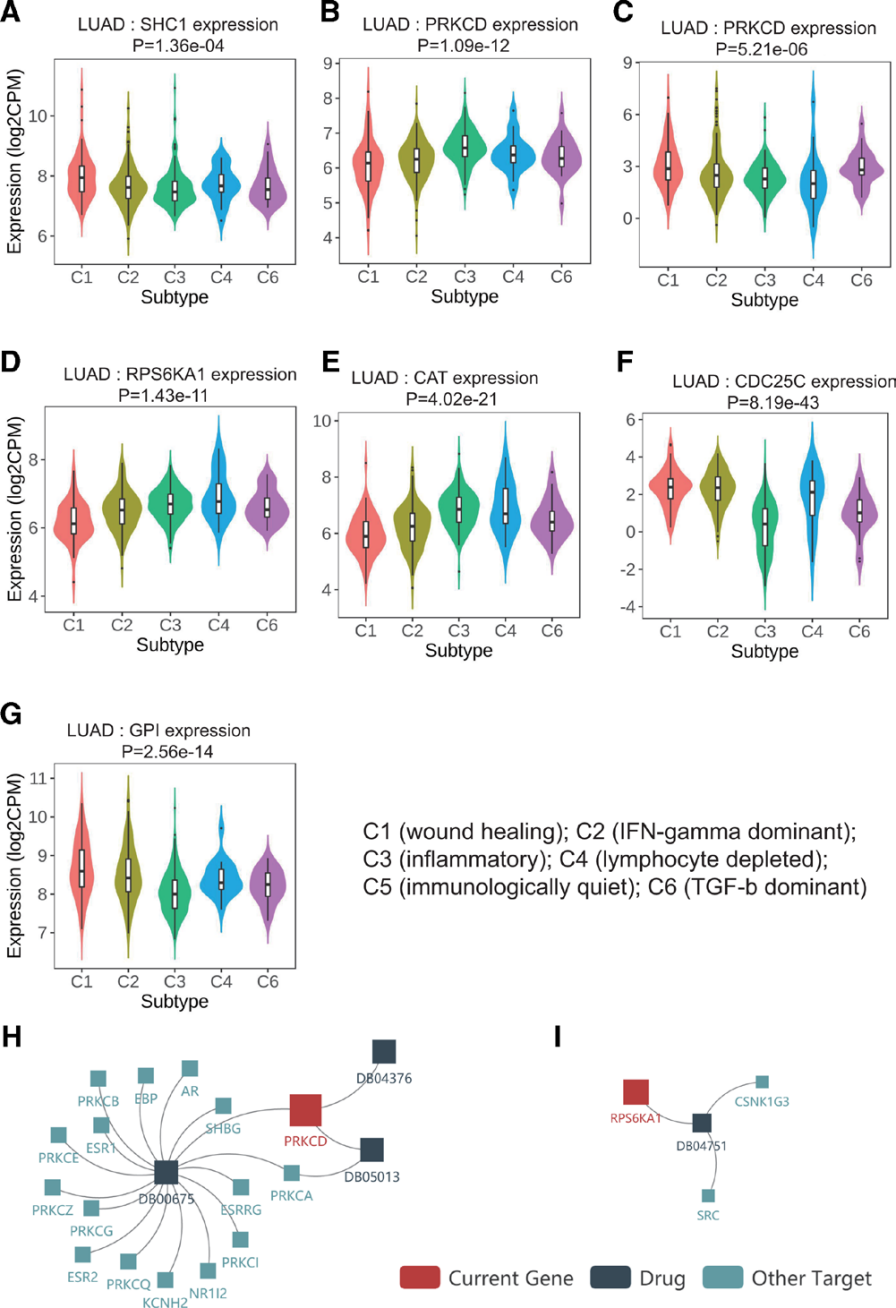
Correlations between gene expression levels with immune subtypes and drug targets in the TISIDB website. (A–G) The relationship between gene expression levels and immune subtypes were statistically significant (all *P*’s < 0.001). C1 (wound healing), C2 (IFN-gamma dominant), C3 (inflammatory), C4 (lymphocyte depleted), C5 (immunologically quiet), and C6 (TGF-b dominant). (H) Targeting *PRKCD*. (I) Targeting *RPS6KA1*. DB04751 = purvalanol A, DB00675 = tamoxifen, DB04376 = 13-acetylphorbol, DB05013= ingenol mebutate.

Additionally, the results from TISIDB platform demonstrated that the expression of all 7 signature genes in LUAD patients was closely linked to 6 immune subtypes (Fig. 7A–G, all *p*’s < 0.001), including wound healing, IFN-gamma dominant, inflammatory, lymphocyte depleted, immunologically quiet, and TGF-b dominant. We were very surprised to learn that *PRKCD* (Fig. 7H) and *RPS6KA1* (Fig. 7I) have been used as targets in drug studies.^[31,32]^ For example, tamoxifen, which targets *PRKCD*, has been used to treat advanced breast and ovarian cancers.^[33]^ Furthermore, we found through the DrugBank database (https://go.drugbank.com/) that Fostamatinib has been used as an inhibitor of RPS6KA1 for the treatment of chronic immune thrombocytopenia.

## 4. Discussion

A large number of previous studies have demonstrated the potential of multiple members of the CXC chemokine and its receptor family as novel immunotherapeutic targets and prognostic biomarkers for a variety of tumors. For example, Tian, H. et al used bioinformatics to identify some members of the CXC chemokine family, CXCL1, CXCL4, CXCL7, and CXCL8 with low expression levels and CXCL12, CXCL14, and CXCL16 with high expression levels had longer OS in LUAD patients.^[34]^ Li, Y. et al analyzed the prognostic and medical value of 17 members of the CXC chemokine family in head and neck squamous cell carcinoma (HNSCC) using multiple public databases, and their results suggested that CXCL1, CXCL2, CXCL3, CXCL8, and CXCL12 may serve as new prognostic markers and treatment targets for HNSCC patients.^[35]^ Spaks et al followed 54 NSCLC patients who underwent radical surgery for up to 6 years and found significantly lower concentrations of CXCL4 and CXCL5 and significantly higher concentrations of CXCL7 in the peripheral blood of the patients. Specifically, only CXCL1 changed in the peripheral blood of patients in the tumor recurrence group. Their study provides further evidence of the immunoediting theory.^[36]^ Furthermore, Qiao, B. and Cong, Z. et al found that high expression of CXCR2 and CXCR4 may serve as indicators of poor prognosis in patients with pulmonary non-small cell carcinoma.^[37,38]^ However, apart from members of the CXC chemokine and its receptor family, there are so many other genes that are closely related to them, and there are few studies on the tumorigenic and developmental processes in which they are involved. No one has previously used chemokine-related genes to construct prognostic signatures and thus to assess the prognosis and immune infiltration status of tumor patients. The construction of chemokine and its receptor-related gene signatures will allow us to better study the powerful functions of chemokines. In our study, we examined the expression of CXCR-associated genes in LUAD tumor tissues as well as their connection to OS. First, we constructed and integrated a novel prognostic gene signature consisting of 7-CXCR related genes, which we then verified using an external dataset (GEO dataset). Functional enrichment analysis demonstrated that cell migration-related pathways were enriched. Finally, we used the TISIDB platform to further analyze the functions of the 7 genes.

The chemokine-receptor system coordinates human cell migration, and perturbations of this system lead to inflammation and cancer; so they have been extensively studied as treatment targets.^[7,10]^ However, their relevance with respect to OS in LUAD patients remains unclear. Most of the CXCR-related genes (62%) were differentially expressed between tumor specimens and neighboring normal specimens, with 13 relevant to OS following univariate Cox regression analysis (*P* < .001). These findings strongly showed that CXCR was involved in the development of LUAD and that CXCR-related genes may be used to set up a prognostic gene signature.

Our prognostic gene signature presented here consisted of 7 CXCR-related genes (*SHC1*, *PRKCD*, *VEGFC*, *RPS6KA1*, *CAT*, *CDC25C*, and *GPI*), and it was an independent predictor of prognosis for LUAD patients. Several previous studies have reported that the *SHC1* gene produces 3 isoforms, each with different functions and subcellular locations. Though all are signal transduction adapter proteins, the longest (p66Shc) is involved in life span regulation and influences reactive oxygen species (ROS). The other 2 isoforms, p52Shc and p46Shc, can activate the GRB2/SOS complex, thereby allowing activated receptor tyrosine kinases to communicate with the Ras pathway.^[39,40]^ Notably, tyrosine kinase signaling within cancer cells is important in the construction and regulation of an immunosuppressive microenvironment.^[41]^ The protein encoded by *PRKCD* is activated by diacylglycerols and acts as both a tumor suppressor and as a positive cell cycle regulator. This protein has the ability to either positively or negatively control apoptosis. As a result, it has great potential as a therapeutic target.^[42,43]^ *VEGFC* is well-known for encoding proteins that promote angiogenesis and endothelial cell growth, and it can influence vascular permeability,^[44]^ a process closely related to tumor cell metastasis. *RPS6KA1* has 2 distinct kinase catalytic domains that can phosphorylate many substrates. The activity of its protein is linked to cell proliferation and differentiation, and it can affect cancer cells.^[45]^ Typically, malignant cells exhibit elevated ROS levels and alterations in antioxidant molecules compared to normal cells. The leading endogenous oxidative stress promotes tumor proliferation by affecting genetic instability, cell growth and angiogenesis.^[46]^ CAT gene can encode catalase, which is a key antioxidant for the body to resist oxidative stress, which means that CAT gene plays an important role in preventing cancer metastasis. In mammalian cells, *CDC25C* is primarily a nuclear protein, and it is believed to also inhibit p53-induced growth arrest.^[47]^ CDC25 phosphatases can function as a node, whereby they receive mitogenic signals and facilitate the progression of the cell cycle. Because of its critical function in cell cycle regulation, *CDC25* is an excellent target for cancer treatment.^[47,48]^ GPI anchor attachment 1 (GPAA1) can attach the GPI anchor to the ER protein and has been reported to promote EGFR-ERBB2 dimerization, which is advantageous to cancer metastasis and progression, as it promotes the expression of cancer-associated GPI-anchored proteins and provides a more stable platform for EGFR-ERBB2 dimerization (lipid rafts).^[49]^

Although chemokines and their receptors have long been studied, few reports have used their associated genes to build prognostic signatures in LUAD patients. Here, we constructed a 7-gene signature for LUAD patients and evaluated its performance and validity using an independent dataset. Patients were classified into high- and low-risk categories according to the median risk score of the gene signature. The accuracy of this classification was confirmed in both the TCGA and GEO datasets, as the high-risk category had a shorter OS. We also explored the association of each gene in the signature with survival, immune subtypes, and drug targets in LUAD patients using the TISIDB web tool.

We must highlight several limitations in our study. First, retrospective data from public databases were used to construct and validate our prognostic gene signature. To validate its clinical utility, actual prospective data are needed. Second, an inherent weakness exists when considering only individual markers for a prognostic signature; many important prognostic genes in LUAD may have been precluded. Further, the protein expression levels of these genes have not been experimentally validated.

## 5. Conclusion

In conclusion, our work has identified a novel prognostic gene signature based on 7 CXCR-related genes. In both the derivation and validation datasets, this signature was found to be independently correlated with OS, thereby delivering insight into assessing LUAD prognosis. However, the potential mechanisms underlying the relationship between CXCR-related genes and tumor immunity in LUAD are still ambiguous, and therefore further research is required.

## Acknowledgments

The authors thank all participants and contributors to this study, including all the staff of the Department of Thoracic Surgery, The First Affiliated Hospital of Guangxi Medical University.

## Author contributions

**Conceptualization:** Shikang Li.

**Data curation:** Kun Deng.

**Formal analysis:** Kun Deng, Zhanyu Xu, Junqi Qin.

**Investigation:** Shenghua Lin, Yu Sun.

**Methodology:** Shenghua Lin, Zhanyu Xu, Liqiang Yuan, Fanglu Qin.

**Resources:** Junqi Qin, Tiaozhan Zheng.

**Software:** Liqiang Yuan.

**Validation:** Yu Sun, Jiangbo Wei, Tiaozhan Zheng, Zhiwen Zheng.

**Writing – original draft:** Kun Deng.

**Writing – review & editing:** Fanglu Qin, Shikang Li.
